# Molecular Detection and Identification of Bacterial Pathogens in Qinghai Province, China

**DOI:** 10.3390/pathogens15030305

**Published:** 2026-03-11

**Authors:** Didi Zhang, Yihong Ma, Xinyuan Zhao, Huaixing Yang, Xiuping Li, Guanghua Wang, Yong Hu, Shenghua Tang, Rong Li, Shizhen Li, Yingna Jian, Liqing Ma

**Affiliations:** 1 Qinghai Provincial Key Laboratory of Pathogen Diagnosis for Animal Diseases and Green Technical Research for Prevention and Control, College of Agriculture and Animal Husbandry, Academy of Animal Sciences and Veterinary, Qinghai University, Xining 810016, China; didizhang1007@outlook.com (D.Z.); zhaoxinyuan020501@hotmail.com (X.Z.); 15290028163@139.com (H.Y.); 1997990036@qhu.edu.cn (X.L.); 2011990018@qhu.edu.cn (G.W.); 1991990032@qhu.edu.cn (Y.H.); 2 National Research Center for Protozoan Diseases, Obihiro University of Agriculture and Veterinary Medicine, Obihiro 080-8555, Japan; myhqhhy@163.com; 3 Qinghai Haidong Center for Animal Disease Prevention and Control, Haidong 810600, China; hdxmk8616897@163.com; 4 Animal Husbandry and Veterinary Station of Minhe County, Haidong 810800, China; lirong680822@163.com; 5 Animal Husbandry and Veterinary Station of Menyuan County, Haibei Tibetan Autonomous Prefecture 810399, China; 15897001920@139.com

**Keywords:** ticks, Qinghai–Tibet Plateau, bacterial pathogens, *Rickettsia*, *Brucella*

## Abstract

As a core pastoral region of the Qinghai–Tibet Plateau, Qinghai Province faces substantial threats to livestock production from tick-borne diseases. This study aimed to investigate the prevalence of six bacterial pathogens in dominant tick species from Qinghai Province, to provide baseline epidemiological data for local tick-borne disease surveillance. A total of 1025 questing ticks were collected from key pastoral regions of Qinghai Province during April to May in 2024 and 2025. All ticks were morphologically identified as belonging to 1 family (Ixodidae), 2 genera, and 4 species. *Dermacentor nuttalli* was the dominant species with a relative dominance of 66.83% (685/1025, 95% CI: 63.92–69.61%), followed by *Haemaphysalis qinghaiensis* at 30.83% (316/1025, 95% CI: 28.11–33.69%), *Dermacentor silvarum* at 1.95% (20/1025, 95% CI: 1.27–2.98%), and *Dermacentor niveus* at 0.39% (4/1025, 95% CI: 0.15–1.01%). PCR detection was performed for six target pathogens, with an overall *Brucella* spp. DNA detection rate of 0.78% (8/1025, 95% CI: 0.40–1.53%) and an overall *Rickettsia* spp. detection rate of 16.29% (167/1025, 95% CI: 14.16–18.67%). Statistical analysis showed that the prevalence of *Brucella* spp. and *Rickettsia* spp. differed significantly between the two dominant tick species (Fisher’s exact test/χ^2^ test, all *p* < 0.001). No *Brucella* or *Rickettsia* pathogens were detected in *D. silvarum* and *D. niveus.* Notably, detection of *Brucella* spp. DNA does not confirm the presence of viable bacteria or tick vector competence. This study fills the regional data gap of tick-borne pathogens in Qinghai, and provides reference for the prevention and control of local tick-borne zoonotic diseases.

## 1. Introduction

Ticks are obligate hematophagous arachnids and primary potential vectors for diverse pathogens among humans, livestock, and wildlife [1]. Tick-borne bacterial diseases threaten public health and livestock productivity globally, raising widespread concerns [2,3,4,5]. China harbors 125 tick species (111 Ixodidae, 14 Argasidae), accounting for ~13% of global diversity, with distribution shaped by environmental and climatic factors [5,6,7,8,9].

Qinghai Province, in the northeastern Qinghai–Tibet Plateau (QTP, average altitude >3000 m), is a unique high-altitude pastoral ecosystem and a priority region for emerging/re-emerging zoonotic diseases [10]. Its cold, hypoxic conditions and extensive animal–human interface (yaks and Tibetan sheep as dominant livestock) create favorable niches for ticks and tick-borne pathogens [10,11,12,13]. Thirty-two tick species have been recorded here, with *Haemaphysalis qinghaiensis*, *Dermacentor nuttalli*, and *D. silvarum* as dominant species [11,12,13,14,15,16,17]. These ticks harbor various pathogens, posing risks to local livestock and public health [18,19]—a critical concern given the region’s limited disease surveillance capacity [10].

Notably, brucellosis is endemic in Qinghai Province and has shown an increasing trend in recent years, causing severe economic losses to the livestock industry and public health risks [20]. While *Brucella* spp. DNA has been detected in ticks elsewhere [20], tick-mediated transmission of *Brucella* spp. remains unconfirmed, and no data exist on *Brucella* spp. in Qinghai ticks. Additionally, the QTP’s unique ecological and climatic conditions (e.g., cold temperatures, limited human activity) may alter tick-pathogen interactions, making targeted investigation of tick-borne pathogens here biologically and epidemiologically meaningful.

Five globally important tick-borne zoonotic pathogens—*Anaplasma phagocytophilum*, *Rickettsia* spp., *Borrelia burgdorferi* sensu lato, *Francisella tularensis*, and *Coxiella burnetii*—have been linked to human and livestock illnesses worldwide [5,21,22,23,24]. While *A. phagocytophilum*, *Rickettsia* spp., *B. burgdorferi* sensu lato, and *C. burnetii* have been detected in Qinghai ticks [15], with *A. phagocytophilum* showing a 3.1% DNA detection rate in local tick species [25], data remain fragmented. *F. tularensis*, a high-consequence zoonotic pathogen, has not been reported in Qinghai ticks [26].

Thus, this study systematically investigated their DNA detection rate and distribution in representative Qinghai ticks. This targeted investigation clarifies their epidemiological status in this high-altitude ecosystem, providing epidemiological data for risk assessment.

## 2. Materials and Methods

### 2.1. Tick Collection and Morphological Identification

Tick sampling was conducted during the peak active period of ticks (April to May) in 2024 and 2025. The sampling sites spanned multiple administrative regions of Qinghai Province, including Datong County and Huangyuan County in Xining City; Ledu District, Minhe County, Huzhu County, Xunhua County and Hualong County in Haidong City; Menyuan County and Gangcha County in Haibei Tibetan Autonomous Prefecture; and Jianzha County in Huangnan Tibetan Autonomous Prefecture.

According to the different local landscapes, the flagging method was employed for the collection of questing ticks [15], a total of 1025 questing tick specimens were collected. All tick samples were individually stored in sterile centrifuge tubes filled with 75% ethanol at 4 °C. Prior to downstream experiments, each tick was surface-sterilized by immersion in 75% ethanol, and all ticks were processed and tested individually.

Morphological characteristics of tick specimens, including scutum morphology coxae structure, basis capituli shape, and pulvilli morphology, were observed under a stereomicroscope. The species were determined through morphological comparison with descriptions in *Tick Systematics* [27].

### 2.2. Tick DNA Extraction

Following morphological identification, all collected ticks were washed three times with 75% ethanol to remove surface contaminants. Genomic DNA was extracted from individual adult tick specimens using the Isohair Kit (Nippon Gene, Tokyo, Japan) in strict accordance with the manufacturer’s protocols. The concentration and purity of the extracted DNA were determined using a NanoDrop 2000 ultramicro spectrophotometer (Thermo Fisher Scientific, Waltham, MA, USA), and the DNA samples were stored at −20 °C until downstream molecular analysis.

### 2.3. PCR Detection of Bacterial Pathogens

Primers for the amplification of target genes from the six bacterial pathogens were synthesized by Azenta (Suzhou, China). The primer sequences, target genes, annealing temperatures, and expected amplicon lengths are listed in Table 1.

Conventional PCR was performed in a 10 µL reaction volume containing 2 µL of template DNA, 0.5 µL each of forward and reverse primers (10 µmol/L), 0.075 µL of Taq DNA polymerase (0.5 U) (NEB, Ipswich, MA, USA), 0.2 µL of dNTPs (NEB, Ipswich, MA, USA), 1 µL of 10× PCR buffer (NEB, Ipswich, MA, USA), and 5.725 µL of nuclease-free ddH_2_O.

Nested PCR was performed in a 20 µL reaction volume using a two-step amplification protocol. The first round was performed with outer primers using the same reaction conditions described above. For the second round, 2 µL of a 10-fold diluted first-round product was used as the template with inner primers, and the same PCR conditions were applied, including the primer-specific annealing temperature.

A negative control was included, using nuclease-free ddH_2_O instead of DNA template. Positive controls for *A. phagocytophilum*, *Rickettsia* spp., *C. burnetii*, and *Brucella* spp. were recombinant plasmids containing the target gene fragments cloned into the pMD19-T vector and verified via Sanger sequencing. For *B. burgdorferi* s.l. and *F. tularensis*, synthetic plasmids harboring the target sequences were used.

PCR cycling conditions were as follows: initial denaturation at 95 °C for 5 min; 35 cycles of denaturation at 95 °C for 30 s, primer-specific annealing for 35 s, and extension at 68 °C for 1 min; followed by a final extension at 68 °C for 10 min. Amplification products separated via 1.5% agarose gel electrophoresis, and results were determined based on expected amplicon sizes (Table 1).

### 2.4. Cloning and Sequencing of Positive PCR Amplicons

For each target pathogen, all PCR-positive samples were selected for sequencing and phylogenetic analysis, to ensure the specificity of detection results and avoid non-specific amplification. PCR amplicons from positive samples were purified using the EasyPure Quick Gel Extraction Kit (TransGen Biotech, Beijing, China) and subsequently cloned into *Escherichia coli* DH5α competent cells using the pMD™19-T Vector Cloning Kit (Takara, Shiga, Japan) according to the manufacturer’s instructions. At least three positive clones per sample were selected and submitted to Genewiz (Suzhou, China) for Sanger sequencing.

### 2.5. Statistical Analysis

All statistical analyses were performed using GraphPad Prism 9.0 (GraphPad Software, San Diego, CA, USA). Prevalence rates were calculated on a per individual tick basis, and 95% confidence intervals (95% CIs) for all proportions were determined using the Wilson score method in GraphPad Prism 9.0. The Chi-square test and Fisher’s exact test were used to compare pathogen prevalence between *H. qinghaiensis* and *D. nuttalli* with a *p*-value < 0.05 considered statistically significant.

### 2.6. Sequence and Phylogenetic Analyses

Sequencing results were manually corrected via chromatogram analysis, homologous sequence searches were performed using the Basic Local Alignment Search Tool (BLAST) against the GenBank database (https://blast.ncbi.nlm.nih.gov/Blast.cgi (accessed on 10 September 2025)). Multiple sequence alignment was performed using the ClustalW algorithm integrated in MEGA X software (version 10.2.6) [33,34], with default parameters, and all gapped positions were retained for subsequent analysis. Phylogenetic trees were constructed using the Maximum Likelihood (ML) method with the Kimura 2-parameter (K2) model in MEGA X software. Bootstrap analysis was performed with 1000 replicates, and only values ≥ 70% are shown at the nodes. Based on sequence homology and phylogenetic clustering, the detected pathogens were preliminarily classified at the genus or species level.

## 3. Results

### 3.1. Sample Collection

**Figure 1:**
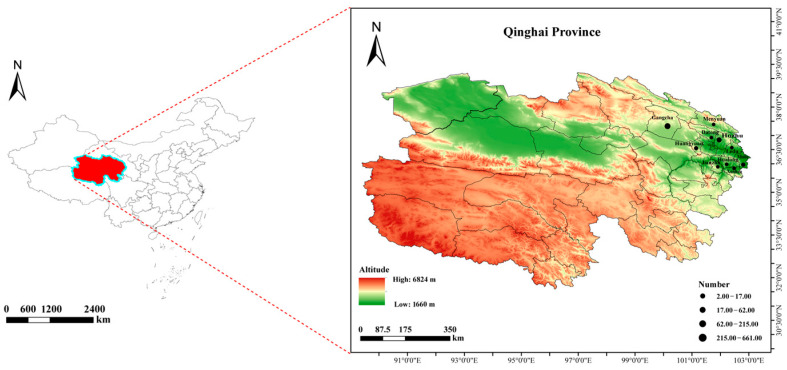
Tick sampling map of selected areas in Qinghai Province. The red area highlights Qinghai Province within China, while the green-to-orange gradient represents the altitude gradient (low: 1660 m to high: 6824 m). Black dots indicate sampling sites, with size proportional to the number of ticks collected.

A total of 1025 questing ticks were collected (April to May) in 2024 and 2025. The geographical distribution and habitat characteristics of all sampling sites are shown in Figure 1. The map highlights all sampling sites, with labels indicating the number of ticks collected and the dominant habitat type at each location. The map was generated using ArcGIS 10.8 software (version 10.7.0.10450).

### 3.2. Morphological Identification of Tick Species

**Figure 2:**
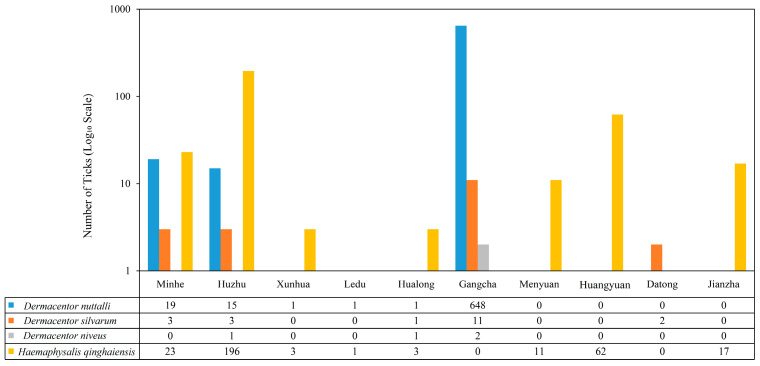
Distribution of tick species across sampling sites in Qinghai Province (2024–2025). The stacked bar chart illustrates the number of ticks per species at each site, with *D. nuttalli* (blue), *H. qinghaiensis* (yellow), *D. silvarum* (orange), and *D. niveus* (gray) represented by distinct colors.

**Figure 3:**
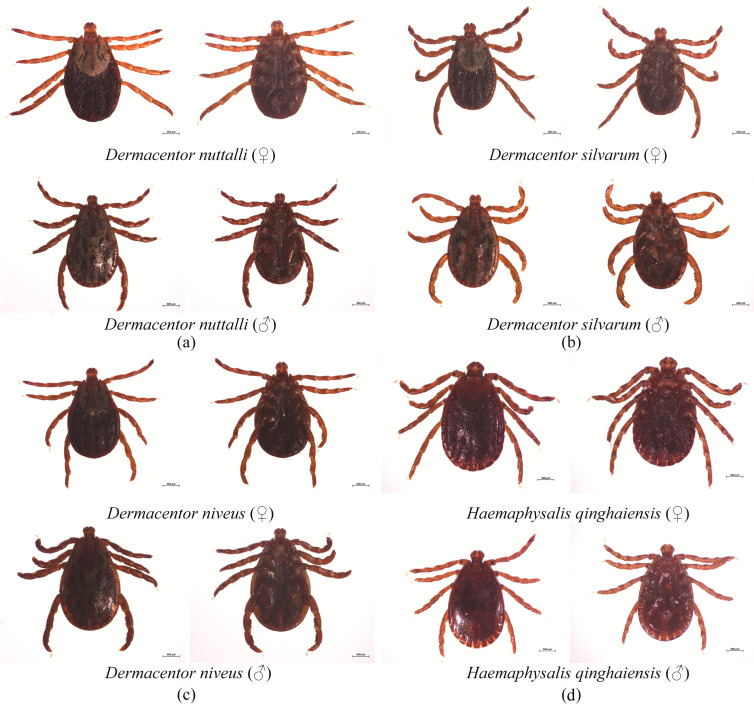
Morphological characteristics of ticks collected from the Qinghai–Tibet Plateau. (**a**) *D. nuttalli*; (**b**) *D. silvarum*; (**c**) *D. niveus*; (**d**) *H. qinghaiensis*.

A total of 803 ticks were collected in 2024, and 222 ticks were collected in 2025. All 1025 questing ticks were adult ticks, and no larvae or nymphs were included. All ticks were morphologically identified and classified into 1 family (Ixodidae), 2 genera (*Dermacentor* and *Haemaphysalis*), and 4 species: *D. nuttalli*, *H. qinghaiensis*, *D. silvarum*, and *D. niveus*. Among these, *D. nuttalli* had the highest relative abundance at 66.83% (685/1025, 95% CI: 63.92–69.61%), followed by *H. qinghaiensis* at 30.83% (316/1025, 95% CI: 28.11–33.69%), *D. silvarum* at 1.95% (20/1025, 95% CI: 1.27–2.98%), and *D. niveus* at 0.39% (4/1025, 95% CI: 0.15–1.01%). The geographical distribution of these tick species across sampling sites (e.g., Gangcha, Menyuan, Huangyuan, etc.) is presented in Figure 2, in which *D. nuttalli* was the dominant species in Gangcha (648 ticks), while *H. qinghaiensis* was primarily concentrated in Huzhu (196 ticks) and Hualong (3 ticks). *D. silvarum* and *D. niveus* were detected in low quantities across scattered sites. The morphological characteristics of *H. qinghaiensis*, *D. nuttalli*, *D. silvarum*, and *D. niveus* are illustrated in Figure 3.

### 3.3. PCR Detection Results

All prevalence rates reported in this study were calculated on an individual tick basis, with no pooled sampling or site-based calculations employed. PCR amplification using pathogen-specific primers (Table 1) revealed the presence of *Brucella* spp. and *Rickettsia* spp. in the tested tick population, with detailed detection outcomes summarized in Table 2. The overall prevalence of *Brucella* spp. in tested ticks was 0.78% (8/1025, 95% Wilson CI: 0.40–1.53%), with all positive samples detected in *H. qinghaiensis* (2.53%, 8/316, 95% CI: 1.29–4.92%); no *Brucella* spp. were detected in *D. nuttalli* (0/685, 95% CI: 0.00–0.56%), and the prevalence difference between the two tick species was statistically significant (Fisher’s exact test, *p* < 0.001). For *Rickettsia* spp., the overall prevalence was 16.29% (167/1025, 95% CI: 14.16–18.67%), with a prevalence of 7.59% (24/316, 95% CI: 5.16–11.05%) in *H. qinghaiensis* and 20.88% (143/685, 95% CI: 18.00–24.08%) in *D. nuttalli*; the difference between the two tick species was statistically significant (χ^2^ = 27.41, df = 1, *p* < 0.001).

No amplicons were detected for *B. burgdorferi sensu lato*, *A. phagocytophilum*, *C. burnetii*, or *F. tularensis* in any of the tested ticks, indicating that these pathogens were not detected in the sampled questing tick population.

### 3.4. Sequencing Identification and Phylogenetic Analysis of Bacterial Pathogens

**Figure 4:**
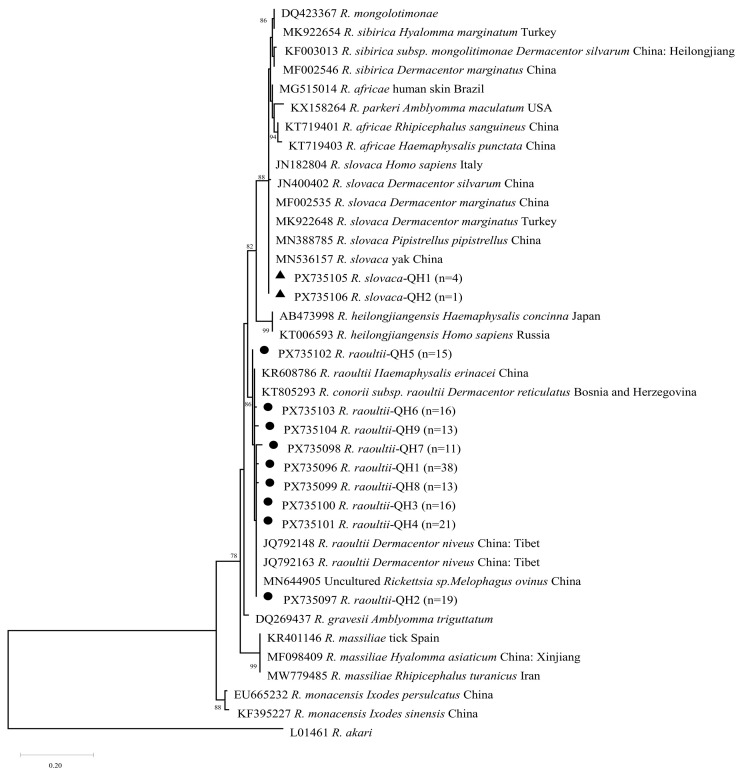
Phylogenetic tree of *Rickettsia* spp. based on *ompA* gene sequences detected in ticks in this study and reference sequences retrieved from the GenBank database. The tree was constructed using the Maximum Likelihood (ML) method with the Kimura 2-parameter (K2) model in MEGA X software. Bootstrap analysis was performed with 1000 replicates, and only values ≥ 70% are shown at the nodes. *Rickettsia akari* (L01461) was used as the outgroup. The tree is drawn to scale, with branch lengths measured in the number of substitutions per site. Black dots (●)/triangles (▲) represent the sequences obtained from ticks in this study; all other sequences are reference strains retrieved from GenBank, with their GenBank accession numbers, species names, host/vector, and geographic origins indicated.

Among the 1025 questing tick DNA samples, two *Rickettsia* species (*R. slovaca* and *R. raoultii*) and *Brucella* spp. were detected via PCR amplification and sequence alignment. The detected *Rickettsia* spp. were divided into two clusters: five *R. slovaca* isolates (3.00%, 5/167) all detected in *H. qinghaiensis*, and 162 *R. raoultii* isolates (97.00%, 162/167), including 143 from *D. nuttalli* and 19 from *H. qinghaiensis*. According to the phylogenetic tree (Figure 4), the *R. raoultii* sequences from this study (GenBank accession numbers: PX735096–PX735104) clustered into a branch with *R. raoultii* isolates from Tibet, China (GenBank accession numbers: JQ792148, JQ792163), with a sequence identity of 99.20% to 100%.

The *R. slovaca* sequences (GenBank accession numbers: PX735105, PX735106) formed a distinct clade alongside the *R. slovaca* reference strain isolated from yaks in Qinghai, China (MN536157) and the strain derived from *Pipistrellus abramus* lung/kidney tissues in Xinjiang, China (MN388785), with sequence identities ranging from 96.97% to 99.39%. These results indicate that the *R. raoultii* strains in this study are closely genetically related to isolates from Tibet, while the *R. slovaca* strains are phylogenetically close to known reference strains from Qinghai and Xinjiang. This implies potential regional genetic continuity of these *Rickettsia* species across the QTP and its surrounding areas.

**Figure 5:**
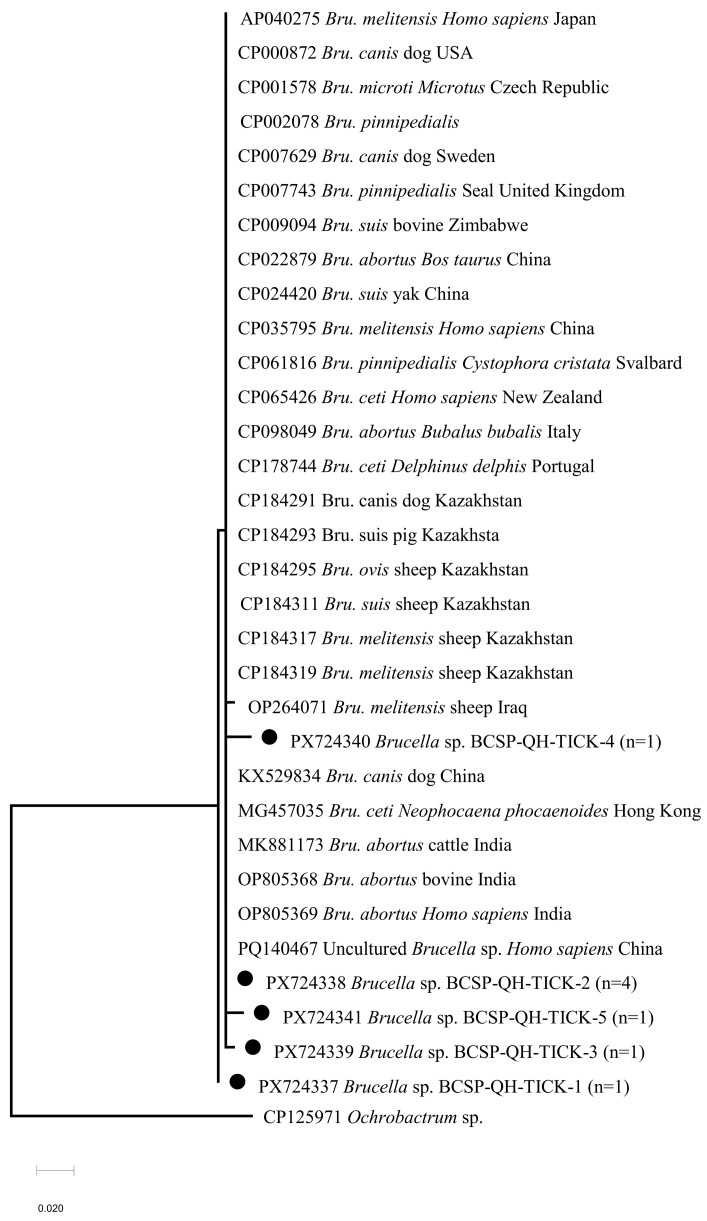
Phylogenetic tree of *Brucella* spp. based on *BCSP31* gene sequences detected in ticks in this study and reference sequences from GenBank. The tree was constructed using the Maximum Likelihood (ML) method with the Kimura 2-parameter (K2) model in MEGA X software. Bootstrap analysis was performed with 1000 replicates, and only bootstrap values ≥ 70% are shown at the nodes. *Ochrobactrum* sp. (CP125971) was used as the outgroup to root the tree, as it is a closely related taxon to the genus *Brucella* and has been widely used for rooting *Brucella* phylogenetic trees in previous research. The tree is drawn to scale, with branch lengths measured in the number of nucleotide substitutions per site. Black dots (●) represent the *Brucella* sequences identified in ticks from this study; all other sequences are reference strains retrieved from GenBank, with their GenBank accession numbers, species names, host/vector, and geographic origins indicated.

Eight *H. qinghaiensis* specimens were positive for *Brucella* spp., corresponding to the overall prevalence of 0.78% (8/1025). Phylogenetic analysis of *Brucella* spp. (Figure 5) revealed that four sequences obtained in this study (GenBank accession numbers: PX724337–PX724340) shared >99.0% nucleotide identity with *Brucella melitensis* isolated from aborted sheep milk in Iraq (OP264071). No *Brucella* spp. were detected in *D. nuttalli*, *D. silvarum*, or *D. niveus*.

## 4. Discussion

The Qinghai–Tibet Plateau is a unique high-altitude pastoral ecosystem with high ungulate diversity and considerable tick activity [35,36,37]. In the present study, questing ticks collected from 10 regions in Qinghai Province were identified morphologically and subjected to molecular screening for six tick-borne bacterial pathogens. Our results revealed the presence of *Rickettsia* spp. and *Brucella* spp. in these ticks, providing baseline epidemiological data for tick-borne pathogens in this understudied high-altitude area.

In total, 4 hard tick species of 2 genera (*Dermacentor* and *Haemaphysalis*) within the family Ixodidae were identified, among which *D. nuttalli* (66.83%) and *H. qinghaiensis* (30.83%) were the dominant species. This is consistent with the conclusion of Ma et al. [12,15,38] that *D. nuttalli* and *H. qinghaiensis* are the dominant tick species in Haidong, Qinghai, further confirming that these two tick species dominate the grassland ecosystem in Qinghai. *D. silvarum* (1.95%) and *D. niveus* (0.39%) were present at extremely low relative abundances, which is likely closely related to the sampling environment. Notably, potential bias may arise from the inconsistent geographic coverage and sample size across the two sampling years. All ticks collected in this study were unfed adult questing ticks. The sampling sites in this study were predominantly natural grasslands and artificial pastures, while *D. silvarum* favors mixed coniferous and broad-leaved forests or shrub habitats [11,39,40,41], which may explain the low capture rate in eastern Qinghai. *D. niveus* is mainly distributed in arid and semi-arid grasslands [42,43,44], and the relatively high humidity at our sampling locations likely constrained its survival. Geographically, *D. nuttalli* was collected at high densities across both eastern and western regions of Qinghai, as this species exhibits strong tolerance to low temperatures and starvation, and parasitizes multiple hosts including yaks and sheep [11,15,17,45]. In contrast, *H. qinghaiensis* was primarily concentrated in eastern Qinghai, consistent with previous reports [8,11,13,15], and demonstrates broader tolerance to temperature and humidity variation, completing its life cycle by parasitizing small mammals during the nymphal stage [15,18,46], which facilitates its rapid spread in agro-pastoral ecotones. The high dominance of *D. nuttalli* and *H. qinghaiensis* stems directly from their robust ecological adaptability. Both tick species have been confirmed to be vectors for transmitting various pathogens such as *Anaplasma* and *Theileria* [47,48,49,50].

The high prevalence of *Rickettsia* spp. detected in questing adult ticks in this study highlights the extensive circulation of spotted fever group rickettsiae (SFGR) in Qinghai Province. The marked dominance of *R. raoultii* is likely linked to its strong ecological compatibility with *D. nuttalli*, a tick species that is widely distributed across Qinghai’s grasslands. This association indicates that *D. nuttalli* may act as a natural carrier of *R. raoultii* in the sampled regions, thereby posing a sustained risk to both livestock (e.g., yaks and Tibetan sheep) and pastoral herders, who represent a high-risk population for SFGR exposure. Since only adult ticks were included in the present study, the infection status and potential role of other tick developmental stages in rickettsiae transmission remain to be explored in future investigations. Notably, the dominant *Rickettsia* species associated with *D. nuttalli* varies geographically: *R. sibirica* is predominant in the Inner Mongolia grasslands [6,51,52,53,54], while multiple SFGR species co-circulate in the border regions of Yunnan Province [55,56,57], underscoring the influence of regional ecological and biogeographical factors on pathogen composition. The high nucleotide homology (99.20–100%) between *R. raoultii* isolates from eastern Qinghai and Tibet [58] supports potential dissemination of this pathogen across the QTP, providing new insights into the evolution and spread of SFGR. The prevalence of *Brucella* spp. DNA in this study was 0.78%, with all positive samples detected exclusively in *H. qinghaiensis.* This finding aligns with the observations of Ma et al. [59,60,61], who reported that 16 tick species are capable of harboring *Brucella* spp. Notably, the *Brucella* DNA positive rate in our study was lower than those reported in previous tick-related surveys, which may be related to our use of questing ticks rather than feeding ticks collected from hosts. Phylogenetic analysis confirmed that the eight *Brucella* sequences obtained in this study belong to the genus *Brucella*. However, as we only detected *Brucella* spp. using the BCSP31 gene, additional testing (e.g., AMOS PCR assay [62]) is required for definitive species identification in the future. A previous study detected *Brucella* spp. in *H. qinghaiensis* collected from goats and sheep in Menyuan County, Qinghai Province [63], and the detected *Brucella* DNA may have originated from residual blood meals of previous hosts rather than from bacterial colonization in the tick. The present study is the first to detect *Brucella* spp. DNA in questing *H. qinghaiensis* collected from Huzhu County, Qinghai Province. However, whether *H. qinghaiensis* plays an active role in the maintenance and transmission of *Brucella* spp. remains to be elucidated.

Given that sheep and cattle are the dominant livestock in Qinghai’s pastoral systems, we speculate that these *Brucella* strains may have been introduced via livestock trade and movement, consistent with the global spread of brucellosis through transboundary animal transport. Importantly, the detection of *Brucella* DNA in ticks does not confirm the presence of viable bacteria, nor does it verify the biological vector competence or transmission capacity of ticks for *Brucella* spp. Ticks are suspected to be mechanical or incidental carriers, as *Brucella* does not have a well-characterized classical tick-borne life cycle [59,60,64,65]. Our study only confirmed the presence of *Brucella* nucleic acid in questing ticks, and the biological transmission competence of ticks for *Brucella* has not been verified. Future controlled experimental infection studies are therefore warranted to clarify the vector potential of ticks in *Brucella* transmission.

It is important to acknowledge the limitations of the present study, which should be considered when interpreting the results. First, regarding tick sample processing, ticks in this study were only washed with ethanol, which is insufficient to remove residual host blood or environmental DNA and may potentially lead to false-positive PCR results. Although all collected ticks were unfed questing ticks with no recent blood-feeding behavior, reducing the risk of interference from residual host blood DNA, this limitation cannot be completely ruled out. Second, there are key conceptual distinctions that need to be clarified: DNA detection does not equate to the presence of viable bacteria, as our molecular screening only confirmed the presence of pathogen nucleic acids but did not verify the viability of the bacteria; the detection of pathogens in ticks does not mean that ticks have vector competence, as vector competence requires evidence of pathogen colonization, replication, and transmission in ticks, which were not evaluated in this study; and the detection rate of pathogens does not directly reflect transmission risk, as transmission risk is also affected by factors such as tick feeding behavior, pathogen viability, and host exposure probability. These limitations highlight that the results of this study should be interpreted cautiously, and the inferences about pathogen transmission and tick vector role need to be supported by further experimental evidence.

No positive detections were obtained for *B. burgdorferi* sensu lato, *A. phagocytophilum*, *C. burnetii*, or *F. tularensis* in the tested questing ticks. This absence likely reflects a combination of ecological, temporal, and methodological factors. Specifically, *B. burgdorferi sensu lato* relies on *Ixodes persulcatus* and rodent reservoirs [66,67], which are scarce in the grassland-dominated sampling areas. In addition, the peak activity period for *A. phagocytophilum*-infected ticks typically occurs between June and July [68,69], whereas our sampling was mainly conducted in April and May. Finally, low pathogen loads in individual ticks, particularly for *F. tularensis* [32,70,71,72], may have reduced the sensitivity of our single-tick PCR assay. Pooled-tick testing may therefore improve detection of low-prevalence pathogens in future studies.

Collectively, the findings of this study provide baseline molecular data and improve our understanding of the distribution of tick-borne pathogens in the surveyed regions of Qinghai Province, which may serve as a reference for regional veterinary and public health planning in high-altitude pastoral areas. Specifically, given the wide distribution of *D. nuttalli* and the high detection rate of *R. raoultii*, continued monitoring of this pathogen in ticks, livestock, and humans could be considered in regions such as Minhe and Gangcha. Meanwhile, awareness of tick exposure and basic personal protective precautions may be warranted for high-risk groups including pastoral herders and livestock veterinarians. In eastern Qinghai’s agropastoral ecotones where *H. qinghaiensis* is present, continued surveillance of *Brucella* in local livestock and human populations may help to facilitate risk assessment. Furthermore, our data confirm that April to May is the peak active period for both dominant tick species (*D. nuttalli* and *H. qinghaiensis*). These observations highlight the importance of enhanced tick control and monitoring during this period, which may contribute to the prevention and control of tick-borne diseases in the region.

## Figures and Tables

**Table 1:** Primer sequences and reaction conditions for the six target bacterial pathogens.

Species	Target Gene	Primer Sequences (5′–3′)	Amplicon Size (bp)	Annealing Temperature (°C)	Reference
*Rickettsia* spp.	*ompA*	F: GCTTTATTCACCACCTCAAC	212/209	55	[28]
		R: TRATCACCACCGTAAGTAAAT			
*B. burgdorferi* sensu lato	*16S rRNA*	F: GAGGCGAAGGCGAACTTCTG	622	60.2	[15]
		R: CTAGCGATTCCAACTTCATGAAG			
*Brucella* spp.	*BCSP31*	F: TGGCTCGGTTGCCAATATCAA	223	61	[29]
		R: CGCGCTTGCCTTTCAGGTCTG			
*A. phagocytophilum*	*16S rRNA*	F1: CACATGCAAGTCGAACGGATTATTC	932	55	[30]
		R1: TTCCGTTAAGAAGGATCTAATCTCC			
		F2: AACGGATTATTCTTTATAGCTTGCT	546/565		
		R2: GGCAGTATTAAAAGCAGCTCCAGG			
*C. burnetii*	*htpB*	F1: GCGGGTGATGGTACCACAACA	501	57	[31]
		R1: GGCAATCACCAATAAGGGCCG			
		F2: TTGCTGGAATGAACCCCA	325	52	
		R2: TCAAGCTCCGCACTCATG			
*F. tularensis*	*16S rRNA*	F: CTGTATCATCATTTAATAAACTGCTG	400	60	[32]
		R: AGTGCCATGATACAAGCTTCCCAA			

Footnotes: F, forward primer; R, reverse primer.

**Table 2:** PCR detection results of target pathogens in collected ticks.

Species	Target Gene	Positive Ticks (*n*)	Positive Rate (%)	Fragment Size (bp)	Detection Result
*Brucella* spp.	*BCSP31*	8	0.78	223	Detected
*Rickettsia* spp.	*ompA*	167	16.29	212/209	Detected
*B. burgdorferi* sensu lato	*16S rRNA*	0	0	622	Not detected
*A. phagocytophilum*	*16S rRNA*	0	0	932/546	Not detected
*C. burnetii*	*htpB*	0	0	501/325	Not detected
*F. tularensis*	*16S rRNA*	0	0	400	Not detected

## Data Availability

Sequence data that support the findings of this study have been deposited in the GenBank database (https://www.ncbi.nlm.nih.gov/genbank/ (accessed on 10 September 2025)) under accession numbers PX724337–PX724341 and PX735096–PX735106.
